# Mediastinal pancreatic pseudocyst with isolated thoracic symptoms: a case report

**DOI:** 10.1186/1752-1947-2-180

**Published:** 2008-05-27

**Authors:** Robert Drescher, Odo Köster, Carsten Lukas

**Affiliations:** 1 Institute of Diagnostic and Interventional Radiology and Nuclear Medicine, Ruhr-University Bochum, St Josef University Hospital, Bochum, Germany

## Abstract

**Introduction:**

Mediastinal pancreatic pseudocysts represent a rare complication of acute or chronic pancreatitis.

**Case presentation:**

A 55-year-old man with a history of chronic pancreatitis was admitted with intermittent dyspnea, dysphagia and weight loss. Chest X-ray, computed tomography and magnetic resonance imaging revealed a large paracardial pancreatic pseudocyst causing cardiac and esophageal compression.

**Conclusion:**

Mediastinal pancreatic pseudocysts are a rare complication of chronic pancreatitis. These pseudocysts may lead to isolated thoracic symptoms. For accurate diagnostic and therapy planning, a multimodal imaging approach is necessary.

## Introduction

Pseudocyst formation is a common complication of chronic pancreatitis. Usually, these cysts are located inside and around the pancreas, and most often arise due to leakage of pancreatic secretions into surrounding tissues. In some cases the connection between the cyst and the pancreas is not evident on computed tomography (CT) or magnetic resonance imaging (MRI). Rarely, pancreatic pseudocysts can extend to the mediastinum [1,2]. They may lead to pleural or pericardial effusion, cardiac compression due to mass effect and dysphagia [3,4].

We report the case a patient with a history of ethanol-induced chronic pancreatitis suffering from intermittent dyspnea and difficulties in swallowing solid foods. Imaging revealed large cystic lesions in the posterior mediastinum and upper abdomen. No symptoms of active pancreatitis were evident at initial admission.

## Case presentation

**Figure 1:**
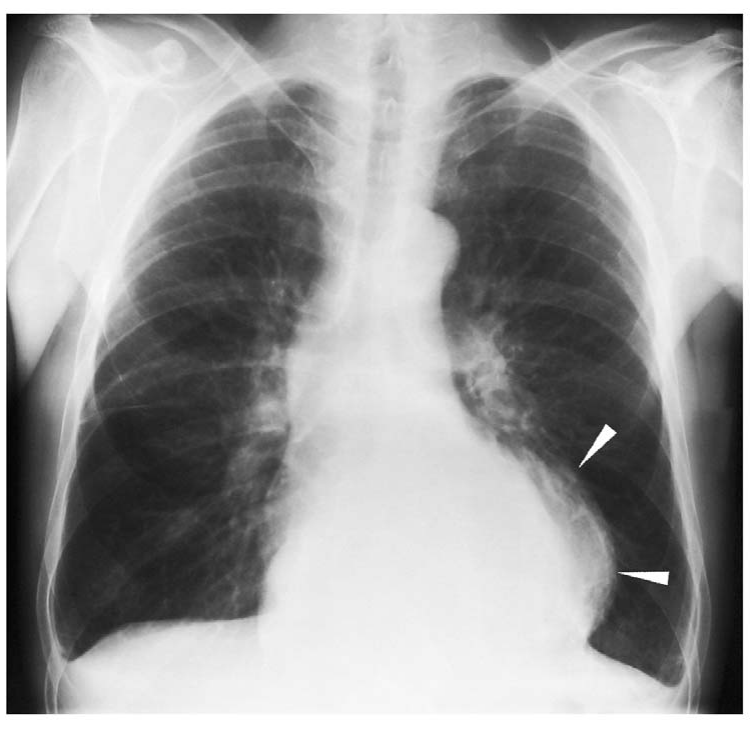
**Chest X-ray on admission**. Initial examination showed an intrathoracic mass overlying the left margin of the heart (arrowheads). No interstitial pulmonary edema was noted. Small pleural effusions are shown.

A 55-year-old man had a history of alcoholic chronic pancreatitis with intermittent acute exacerbations over the last 6 years. On admission, he described recurrent mild-to-moderate dyspnea after exercise and problems in swallowing solid food. He had lost 5 kg in weight during the last 2 months as a result. Clinical examination was inconclusive; laboratory investigations showed no sign of acute pancreatitis exacerbation. Serum amylase and lipase were within the normal range. On chest X-ray, a semitransparent intrathoracic mass adjacent to the heart as well as small bilateral pleural effusions were noted (Figure 1). The lung structure appeared normal. In view of the weight loss and with the differential diagnosis of neoplasm in mind, CT of the chest and upper abdomen was suggested.

**Figure 2:**
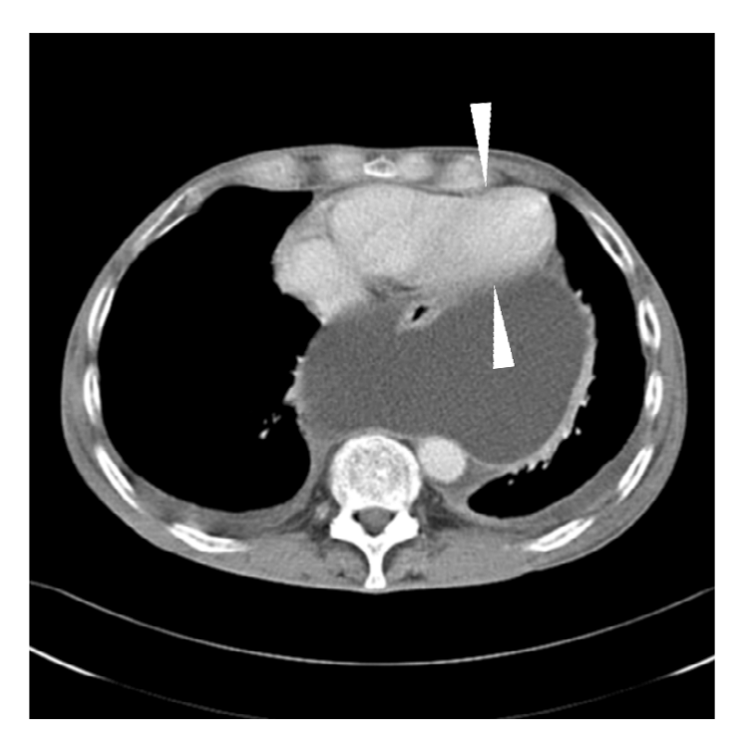
**Contrast-enhanced computed tomography scan of the chest-abdomen**. A large cystic lesion is compressing the heart, predominantly the left ventricle (arrowheads).

**Figure 3:**
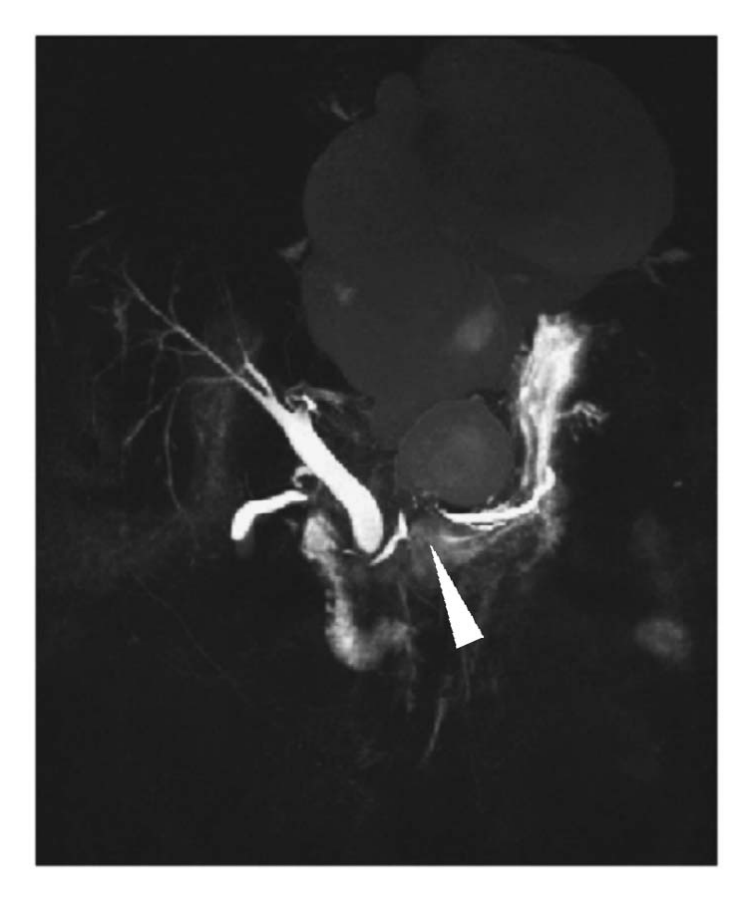
**T2-weighted coronal magnetic resonance imaging of the upper abdomen and magnetic resonance cholangiopancreatography**. There is communication between mediastinal and abdominal pseudocysts through the esophageal hiatus. High-grade ductal stenosis (arrowhead) is shown, but only a slight widening in the pancreatic body and tail.

Contrast-enhanced CT was performed on a 16-slice scanner (slice thickness 5 mm, collimation 16 × 1.5 mm, 100 ml iodinated contrast medium was given intravenously) and revealed multiple cystic lesions extending from the pancreatic head and/or body to the upper abdomen and into the lower mediastinum. The size of the mediastinal cyst was 14.5 × 12 × 16 cm. It was shown by multiplanar reconstructions that all of the lesions were communicating. The esophagus was partially surrounded by large cysts in the retrocardial and hiatal regions, which compressed the left ventricle (Figure 2). A further examination with magnetic resonance cholangiopancreatography (MRCP) showed the cystic structure with a small contact area to the pancreatic tissue and a high-grade stenosis of the pancreatic duct with only moderate dilation up to 6 mm of the distal pancreatic duct (Figure 3). A dedicated contrast-enhanced MRI examination of the pancreas in the same session showed atrophy and postinflammatory tissue changes. No signs of acute inflammation or neoplasm were evident.

Endoscopy combined with endosonography and endoscopic retrograde cholangiopancreatography confirmed the pancreatic duct stenosis and dilatation without communication of the ductal system to the pseudocysts. The stenosis could not be crossed with a guidewire. A small intrapancreatic mass at the site of the stenosis was suspected from endoscopic ultrasound and tissue elastography results. Endoscopic drainage of the cysts was not performed because a transgastric approach to the cysts was not possible. The patient, therefore, underwent surgery. Cysts received external drainage through an abdominal access. Analysis of the cystic fluid demonstrated high levels of amylase (8678 IU/liter) and lipase (37,953 IU/liter). A malignancy was not ruled out by imaging, so part of the pancreas with the stenosis was resected and a side-to-side pancreaticojejunostomy was done. Histology showed postinflammatory changes with no evidence of a neoplasm. Laboratory values of the drained fluid were consistent with pancreatic juice with no evidence of infection.

Follow-up CT after 6 days revealed nearly complete resolution of the pseudocysts. The external drainage was removed accordingly from the asymptomatic patient.

## Discussion

Mediastinal pancreatic pseudocyst was first described in 1951 [5], and it remains a rare complication of pancreatitis. Ethanol-induced pancreatitis is responsible for the majority of cases in adults. Furthermore, post-traumatic occurrence has been described [6]. In general, pseudocysts appear in chronic pancreatitis in the absence of a recent attack of acute pancreatitis, but they may develop after an episode of an acute attack [2,6-10]. Pathophysiologically, mediastinal pseudocysts develop after rupture of the pancreatic duct posteriorly into the retroperitoneal space. In most cases the pancreatic fluid enters the mediastinum through the esophageal or aortic hiatus [1,8].

In the majority of reported cases, these cysts were diagnosed in symptomatic patients. Symptoms may include abdominal, chest and/or back pain, dyspnea, cardiac tamponade, dysphagia, odynophagia, cough and weight loss [2,4,6-8,11]. Most patients suffer from pain in the upper abdomen, which together with the patient's history and laboratory findings of pancreatitis, facilitate the correct diagnosis. Pleural effusion is present in the majority of mediastinal pseudocyst cases [2].

The presence of mediastinal pseudocysts in patients without pancreas-related signs and symptoms (pain, serum enzyme elevation) is unusual. In our case, the patient complained of intermittent dyspnea and dysphagia. He could not definitely connect the symptoms with specific physical activities. For diagnosis, CT scans are superior to ultrasound in detecting mediastinal masses. Sometimes chest X-ray can reveal a space-occupying mass in the posterior or middle mediastinum. Newer techniques such as endoscopic ultrasound have been reported to be extremely useful, particularly when a guided fine needle aspiration is also performed [12]. The initial X-ray in our case showed a semitransparent intrathoracic mass in the lower mediastinum, leading to the differential diagnoses of lipoma, fat-containing hernia, or cystic tumor. CT and MRI scans showed a cystic lesion, and the finding of communicating cystic structures in the upper abdomen confirmed the diagnosis of pancreatic pseudocysts.

Primary therapeutic options include surgery with internal or external drainage of the pseudocysts (cystogastrotomy and cystoenterostomy), percutaneous, transpapillary, transgastric and transesophageal endoscopic drainage [1,2,5,6,9]. Transhiatal drainage of mediastinal pseudocysts has been described [10]. Cases with successful medical therapy using somatostatin analog and bromhexine hydrochloride as well as pseudocyst resolution after abstinence from alcohol and parenteral nutrition have been published [7,13,14]. Endoscopy in our patient revealed that the only possible endoscopic approach would be through the esophageal wall. This has been done successfully [15,16], but in view of the suspected intrapancreatic mass in the endoluminal ultrasound examination causing stenosis of the pancreatic duct and the increased risk of transesophageal puncture, a surgical approach was favored. Without these findings and in cases of a stentable stenosis, the less-invasive treatment of the communicating pseudocysts would have been endoscopic nasopancreatic drainage [8].

In view of the results of laparotomy and histology, it could be suspected that postinflammatory changes led to stricture of the pancreatic duct, stenosis and subsequent rupture of the duct into the retroperitoneal space, where over time, the pseudocysts developed and extended through the esophageal hiatus. The communication of the mediastinum and abdominal parts may explain the intermittent nature of the patient's symptoms: levels of cardiac impairment and pressure on the esophagus depend on the intra-abdominal pressure, which causes a shift of fluid into the mediastinal part of the pseudocyst. Since no malignant neoplasm could be found, it is probable that the weight loss of the patient was due to the difficulties in swallowing.

A multimodal approach of multislice CT with multiplanar reformations and three-dimensional MRCP proved to be necessary for the accurate assessment of pancreatitis complication and were important for intervention planning [17]. Nonetheless, a substantial drawback in this case was that the suspected pancreatic neoplasm could not be ruled out by diagnostic imaging.

## Conclusion

Mediastinal pseudocysts are a rare complication of pancreatitis. They may appear in the setting of acute exacerbation of an underlying chronic pancreatitis, but more often present with unspecific symptoms including dyspnea and dysphagia. Our case has illustrated that pseudocysts should be considered as a differential diagnosis in the evaluation of mediastinal masses in a patient with a history of pancreatitis. For accurate diagnosis and therapy planning, a multimodal imaging approach is necessary.

## Abbreviations

CT: computed tomography; MRCP: magnetic resonance cholangiopancreatography; MRI: magnetic resonance imaging.

## Competing interests

The authors declare that they have no competing interests.

## Consent

Written informed consent was obtained from the patient for publication of this case report and any accompanying images. A copy of the written consent is available for review by the Editor-in-Chief of this journal.

## Authors' contributions

All the authors were involved in examination of the patient as well as in writing and reviewing the manuscript.
